# Complete and circularized genome sequences of five nitrogen-fixing *Bradyrhizobium* sp. strains isolated from root nodules of peanut, *Arachis hypogaea*, cultivated in Tunisia

**DOI:** 10.1128/mra.01078-23

**Published:** 2024-05-15

**Authors:** Besma Bouznif, Amira Boukherissa, Yan Jaszczyszyn, Mohamed Mars, Tatiana Timchenko, Jacqui A. Shykoff, Benoît Alunni

**Affiliations:** 1 Université Paris-Saclay, CEA, CNRS, Institute for Integrative Biology of the Cell (I2BC), Gif-sur-Yvette, France; 2 IDEEV—Laboratoire Ecologie, Systématique et Evolution, CNRS, AgroParisTech Université Paris-Saclay, Gif-sur-Yvette, France; 3 Research Unit Biodiversity and Valorization of Arid Areas Bioresources (BVBAA), Faculty of Sciences, Gabès, Tunisia; 4 Université de Picardie Jules Verne, UMRt BioEcoAgro 1158-INRAE, BIOPI, Amiens, France; 5 Université Paris-Saclay, INRAE, AgroParisTech, Institut Jean-Pierre Bourgin (IJPB), Versailles, France; University of Strathclyde, Glasgow, United Kingdom

**Keywords:** symbiosis, nitrogen fixation, *Bradyrhizobium*, peanut, Tunisia

## Abstract

This manuscript reports the complete and circularized Oxford Nanopore Technologies (ONT) long read-based genome sequences of five nitrogen-fixing symbionts belonging to the genus *Bradyrhizobium*, isolated from root nodules of peanut (*Arachis hypogaea*) grown on soil samples collected from Tunisia.

## ANNOUNCEMENT

Legume plants have evolved the capacity to harbor symbiotic nitrogen-fixing soil bacteria within root nodules. This symbiosis provides these plants the ability to grow in diverse habitats and to become major players in agricultural sustainability (1, 2). Bacteria belonging to the genus *Bradyrhizobium* are known as predominant peanut (*Arachis hypogaea*) symbionts (3–6).

In this announcement, we report the complete genome sequence of five bacterial strains isolated from peanut root nodules and belonging to the genus *Bradyrhizobium* based on average nucleotide identity (ANI) value analysis (Table 1). The soils were sampled from five sites in Tunisia (Table 1). Then, Tunisian traditional varieties of peanuts were grown in pots containing the sampled soils for bacterial trapping. After 7 weeks of greenhouse cultivation (28°C, 16 h photoperiod, 160 µmol·m^−2^·S^−1^), nodules were collected and surface-sterilized in 96% ethanol for 1 min, then in 3% sodium hypochlorite solution for 3 min before being rinsed three times with sterile distilled water and individually crushed, plated on yeast extract mannitol (YM) agar medium and grown for 5 to 10 days at 28°C (7, 8). The strains were purified by successive streaking and single-colony picking and then stored in YM medium with 20% (vol/vol) glycerol at −80°C.

**TABLE 1:** Genome features of the five *Bradyrhizobium* sp. strains

Strain characteristic(s)	Data for:				
	*Bradyrhizobium* sp. BEA-2-5	*Bradyrhizobium* sp. BWA-3-5	*Bradyrhizobium* sp. BWC-3-1	*Bradyrhizobium* sp. NDS-1	*Bradyrhizobium* sp. sBnM-33
Sampling site	Béja, Elmarja	Béja, Wechtata	Béjà, Wechtata	Nabeul, Dar Allouch	Ben Gardane
Peanut variety	Arbi	Arbi	Chounfakhi	Siniya	Massriya
No. of reads (post QC)	91,274	118,365	141,671	99,886	80,650
No. of contigs	1	2	1	1	1
No. of genes	7,837	7,729	8,367	7,134	8,642
Genome length (bp)	8,406,336	8,041,125 (chromosome: 7,897,608; plasmid: 143,517)	8,767,305	7,645,890	9,184,954
Coverage (×)	148	188	161	190	146
N50 (bp)	8,406,336	7,897,608	8,767,305	7,645,890	9,184,954
GC content (%)	63.92	62.51	62.92	64.01	61.62
BUSCO score (%)	99.1	99.9	99.5	99.4	99.2
Closest taxonomic assignation—accession (ANI value to closest species [%])	*Bradyrhizobium pachyrhizi—*GCF_029714545.1 (95.89)	*Bradyrhizobium hereditatis—*GCF_020329435.1 (89.14)	*Bradyrhizobium canariense—*GCF_019402665.1 (94.70)	*Bradyrhizobium frederickii—*GCF_004570865.1 (90.13)	*Bradyrhizobium retamae—* GCF_001440415.1 (92.53)
BioSample ID	SAMN37684786	SAMN37684780	SAMN37683990	SAMN37684784	SAMN37684830
SRA ID	SRR26337671	SRR26335727	SRR26335726	SRR26337672	SRR26336054
GenBank ID	CP136629	CP136626- CP136627	CP136625	CP136628	CP136624

**Fig 1:**
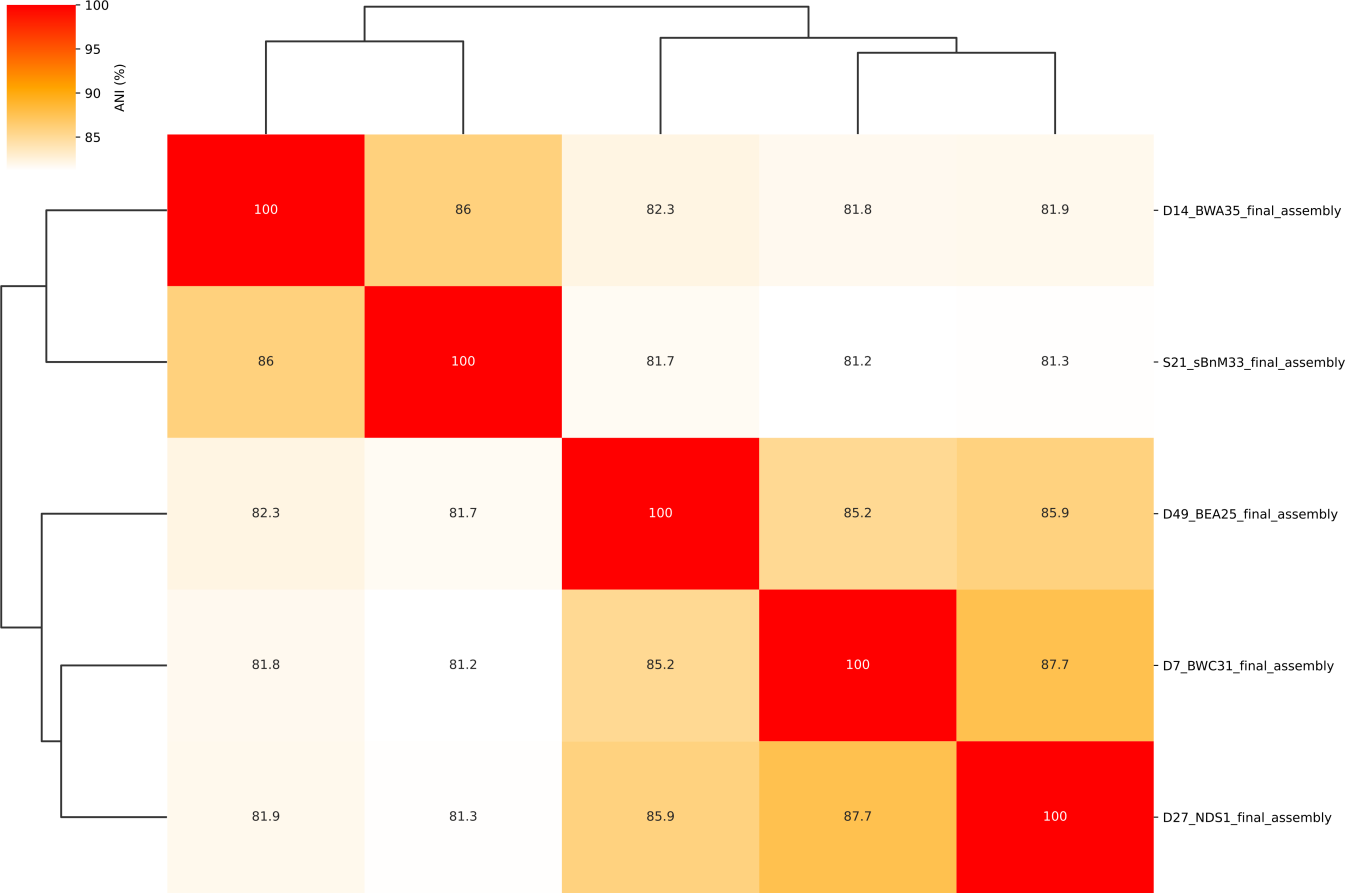
Heatmap of ANI values between the five *Bradyrhizobium* sp. strains. ANI values for pairwise comparisons of these five strains are in the range between 81.2% and 87.7%, below the species threshold of 95%, and therefore appear to belong to five distinct *Bradyrhizobium* species.

Total genomic DNA was extracted from the five strains grown in the YM medium for 4 days at 28°C using the MasterPure complete DNA and RNA Purification Kit (Epicentre). DNA libraries were prepared from intact genomic DNA with the Native Barcoding Kit 24 V14 (SQK-NBD114.24), and sequencing was performed on a MinION flow cell (R10.4.1). Basecalling was performed with the Super Accuracy model in Guppy 5.3.1. Nanopore library adaptors were trimmed using Porechop v2.0.4 (https://github.com/rrwick/Porechop). The read quality was assessed using NanoPlot v1.41.0 (9). The long reads were then assembled using Flye 2.8.3 (10), which generated a single contig assembly for four strains and two contigs for the strain BWA-3-5 (https://doi.org/10.6084/m9.figshare.24547348). The presence of plasmids was determined with plASgraph2 v1.0.0 (11). The assemblies were polished with Medaka v1.7.2 (model r1041_e82_260bps_sup_g632) (https://github.com/nanoporetech/medaka) using the Oxfod Nanopore Technology (ONT) reads to create a consensus sequence. The assemblies of the chromosomes were then rotated with Circlator all at the DnaA gene (12). The plasmid of the strain BWA-3-5 was rotated with Prodigal v2.6.2 at position 73241 (13). The statistics of the assembly were obtained with QUAST v5.2.0 (https://github.com/ablab/quast), and the completeness of the genomes was assessed using BUSCO v5.6.1 (14) (Table 1). Functional annotation of the genomes was performed using the NCBI Prokaryotic Genome Annotation Pipeline v6.3 (15, 16). ANI calculation was carried out using ANIclustermap v1.2.o (Fig. 1)(17).

## Data Availability

The raw reads and complete genomes of the five *Bradyrhizobium* sp. strains were deposited at GenBank under the BioProject no. PRJNA1023532. The BioSample numbers, raw reads, and assembly GenBank accession numbers are given in Table 1.
